# The impact of drug-eluting bead (vs. conventional) transarterial chemoembolization on hepatic fibrosis in treating intermediate or advanced hepatocellular carcinoma

**DOI:** 10.1080/15384047.2023.2166335

**Published:** 2023-02-07

**Authors:** Hao Li, Chao Liang, Donglin Kuang, Guohao Huang, Mengfan Zhang, Pengfei Chen, Qingzhu Zheng, Wenze Xu, Jianzhuang Ren, Xinwei Han, Xuhua Duan

**Affiliations:** Department of Interventional Radiology, the First Affiliated Hospital, Zhengzhou University, Zhengzhou, China

**Keywords:** Hepatocellular carcinoma, drug-eluting bead transarterial chemoembolization, conventional transarterial chemoembolization, hepatic fibrosis, safety

## Abstract

**Objective:**

Limited studies have reported the impact of drug-eluting bead transarterial chemoembolization (DEB-TACE) on hepatic fibrosis in hepatocellular carcinoma (HCC). This study evaluated multiple hepatic fibrosis indicators, aiming to comprehensively compare the influence of DEB-TACE and conventional transarterial chemoembolization (cTACE) on hepatic fibrosis in treating HCC patients.

**Methods:**

Intermediate/advanced HCC patients (N = 121) were divided into the DEB-TACE group (n = 62) and the cTACE group (n = 59) based on their chosen treatment. Serum hyaluronic acid (HA), pro-collagen type-III (PC-III), collagen type-IV (IV-C), and laminin (LN) were detected; aminotransferase to platelet ratio index (APRI) and fibrosis index based on the four factors (FIB-4) were calculated; liver stiffness measurement (LSM) was assessed by real-time shear wave elastography.

**Results:**

HA, PC-III, IV-C, and LN at 1 month after the second TACE and at 12 months after the first TACE were all decreased in DEB-TACE group compared with cTACE group (all *P* < .050). Then, APRI, FIB-4, and LSM were further assessed, which also showed a decreasing trend at aforementioned timepoints in DEB-TACE group compared with cTACE group (all *P* < .050). Additionally, the multivariate logistic regression analysis revealed that DEB-TACE (vs. cTACE) was independently associated with reduced occurrence of severe hepatic fibrosis at 12 months (OR = 0.215, 95%CI: 0.058–0.802, *P* = .022). Concerning the liver function indexes, alanine aminotransferase, aspartate aminotransferase, and total bilirubin after treatment were not different between the two groups (all *P* > .050).

**Conclusion:**

DEB-TACE displays attenuated hepatic fibrosis progression and noninferior tolerance compared to cTACE in treating intermediate- or advanced-stage HCC patients.

## Introduction

Hepatocellular carcinoma (HCC), accounting for over 80% of all liver cancer cases, is a major classification of primary liver malignancy.^1,2^ Currently, HCC remains the fifth most common cancer and the second leading cause of cancer-related death in China in 2020.^3,4^ Etiologically, the occurrence of HCC is associated with multiple risk factors, including hepatitis B virus (HBV) or hepatitis C virus (HCV) infection, excessive alcohol intake, type II diabetes, etc.^5^ To date, great efforts have been made to improve HCC management; for early-stage patients, radical treatment (including surgical resection, liver transplantation, and local ablation) is the primary recommendation.^6,7^ However, intermediate or advanced HCC patients are unsuitable for the aforementioned treatment therapies, and their prognosis is far from satisfactory.^8,9^ Meanwhile, hepatic fibrosis, representing the liver injury degree, correlates with increased recurrence after curative resection; therefore, hepatic fibrosis is another non-ignorable concern for intermediate or advanced HCC patients.^10–12^

Transarterial chemoembolization (TACE) is an embolization therapy that blocks the hepatic artery and delivers chemotherapy drugs to targeted tumor lesions.^13^ TACE has been continuously recommended as the first-line treatment for intermediate-stage HCC patients, and it is even adopted for advanced HCC patients.^14^ Conventional TACE (cTACE) is performed by injecting a mixture of lipiodol-based emulsion plus embolizing agents (such as gelatin sponges and polyvinyl alcohol particles) and cytotoxic drugs into the hepatic feeding artery to treat HCC.^15^ However, some nonignorable shortages of cTACE are observed due to the liquid motility of lipiodol, including the unstable embolization effect, limited loading and release profile, and relatively low drug concentration in the target lesion.^16^

The emergence of drug-eluting bead TACE (DEB-TACE) could overcome the aforementioned shortcomings.^17^ The more favorable efficacy and safety profiles of DEB-TACE treatment (vs. cTACE) have been reported in many studies.^18–20^ For instance, one previous study indicated that DEB-TACE achieves superior treatment response, overall survival (OS), and less liver/systemic toxicity than cTACE in treating intermediate- or advanced-stage HCC patients.^18^ Unfortunately, both cTACE and DEB-TACE might aggravate the impairment of liver tissues (especially through multiple TACE procedures), leading to constant hepatic fibrosis progression.^21^ However, limited evidence supports that DEB-TACE exhibits attenuated liver impairment compared with cTACE.

Hence, this study evaluated multiple hepatic fibrosis indicators (reflected by the serologic and elastography index), aiming to comprehensively compare the impact of DEB-TACE and cTACE on hepatic fibrosis in treating HCC patients.

## Methods

### Patients

This study included 121 newly diagnosed HCC patients who received cTACE or DEB-TACE from October 2019 to September 2020.

The inclusion criteria were as follows: (i) confirmed as HCC by imaging or pathology examinations; (ii) unable or unwilling to undergo surgical treatment; (iii) had at least one measurable lesion; (iv) had no treatment after diagnosis, including liver transplantation, surgical resection, TACE, radiofrequency, microwave, chemical ablation, argon helium knife, ultrasound knife, radiotherapy, systemic chemotherapy, liver cancer-targeted drugs, and immunotherapy; (v) single or sum of 2–3 tumor diameters ≥ 5 cm; (vi) Child-Pugh class A ~ B; (vii) Barcelona Clinic Liver Cancer (BCLC) stage of B ~ C; (viii) aged within 18 ~ 75 years; (ix) Eastern Cooperative Oncology Group performance status (ECOG PS) score of 0 ~ 1 within 1 week before enrollment; (x) tumor occupancy <75% of whole liver; and (xi) life expectancy ≥ 12 weeks.

The exclusion criteria were as follows: (i) diffuse liver cancer; (ii) renal failure, cardiopulmonary failure, or uncorrectable coagulation dysfunction; (iii) gastrointestinal bleeding within 6 months before enrollment or a clear tendency of gastrointestinal bleeding; (iv) portal vein trunk entirely blocked by cancer thrombus; (v) obvious arterial or venous fistula; (vi) systemic condition failure; and (vii) radiofrequency, microwave ablation, or particle implantation during cTACE or DEB-TACE treatment.

The study was approved by the Ethics Committee of The First Affiliated Hospital, Zhengzhou University and registered on the China Clinical Trials Registry (No. ChiCTR-IOR-17012159). All patients signed informed consent forms.

### Data collection

Clinical characteristics were obtained, including age, sex, ECOG PS score, etiology, tumor distribution, largest nodule size, tumor capsule, portal vein invasion, splenomegaly, Child-Pugh class, BCLC stage, and alpha-fetoprotein (AFP).

### Treatment

Treatment (cTACE or DEB-TACE) was performed based on the patient’s disease status and the discussion between doctors and patients. Patients who received cTACE were considered the cTACE group (N = 59); patients who received DEB-TACE were considered the DEB-TACE group (N = 62).

Abdominal enhancement enhanced computed tomography (CT), magnetic resonance imaging (MRI), and other related examinations were performed to clarify the tumor number, tumor size, tumor location, and blood-supplying artery. Then, cTACE or DEB-TACE was carried out. Briefly, the right femoral artery was punctured by a modified Seldinger technique, and then a 5 F catheter was used to perform angiography of the abdominal trunk and superior mesenteric artery to identify the blood-supplying artery. Following that, a microcatheter was catheterized by superselective catheterization. Next, 100 mg of oxaliplatin was injected, and then the microspheres with the contrast agent and emulsions were injected into the blood-supplying artery for the DEB-TACE group and the cTACE group. The subsequent embolization regimen was decided according to the group assignment.

For the DEB-TACE group, 100–300 μm CalliSpheres drug-loaded microspheres (CalliSpheres® Beads CB; Jiangsu Hengrui Medicine Co, Ltd., China) loaded with 60 mg epirubicin or pirarubicin were used for embolization until the staining of the tumor disappeared. If the microspheres were used up, an additional 350 μm-560 μm PVA particles (Hangzhou Alikang Pharmaceutical Technology Co., Ltd., China) were added. The number of TACE in the DEB-TACE group ranged from 1 to 5. For the cTACE group, emulsions of lipiodol (Laboratoire Guerbet, France) and 60 mg epirubicin or pirarubicin were administered for chemoembolization, which was continued until the tumor staining disappeared or deposits of lipiodol appeared in the small branches of the portal vein. If the emulsions of lipiodol were used up, an additional 350–560 μm polyvinyl alcohol (PVA) particles were embolized until the tumor staining disappeared, not exceeding 20 ml of lipiodol in total. The number of TACE in the cTACE group ranged from 1 to 9.

At 6 hours postoperatively, patients underwent cardiac monitoring and symptomatic supportive treatment, such as analgesia, hepatoprotection, and antiemetics. For patients with HBV or HCV infection, oral antiviral drugs were administered postoperatively. At 4–6 weeks after surgery, patients were reviewed by CT or MRI, and if lesions were found to be incompletely necrotic or if *de novo* lesions existed, the operation (cTACE or DEB-TACE) was repeated with the same dose as the first treatment.

### Assessment

Biochemical indexes before TACE, at 1 month after the first TACE, 1 month after the second TACE, and 12 months after the first TACE were obtained, including liver function indexes (alanine aminotransferase (ALT), aspartate aminotransferase (AST), albumin (ALB), total bilirubin (TBIL)), liver fibrosis indexes (hyaluronic acid (HA), pro-collagen type-III (PC-III), collagen type-IV (IV-C), laminin (LN)). Then, the aminotransferase to platelet ratio index (APRI) and fibrosis index based on the four factors (FIB-4) were calculated.^22^ Liver stiffness measurement (LSM) was assessed by real-time shear wave elastography (SWE).^23^ All SWE operations were completed by doctors with 3 years of experience in the use of conventional ultrasound and 2 years of experience in SWE operations. SWE images were acquired at 5 different levels for each patient, and the mean LSM was calculated. Severe hepatic fibrosis was defined as the LSM at 12 months after the first TACE ≥17.5, which was identified by the receiver operating characteristic curve as in a previous study.^24^

Patients received routine follow-ups, and the disease status was recorded. The final date of follow-up was August 1, 2021. Based on follow-up records, progression-free survival (PFS) and OS were imputed. PFS was estimated from the first TACE to disease progression or death from any cause; OS was estimated from the first TACE to death.

Adverse events of patients were evaluated via the Common Terminology Criteria for Adverse Events (CTCAE, 4.0).

### Statistics

Statistics were completed with SPSS 21.0 (IBM Corp., USA). Graphs were made with GraphPad Prism 6.01 (GraphPad Software Inc., USA). Comparison analysis was evaluated with Student’s t test, Chi-square test, or Fisher’s exact test. Factors related to severe hepatic fibrosis were assessed with enter-mode multivariate logical regression model analysis. PFS and OS curves were made with the Kaplan-Meier method and log-rank test. *P* < .050 was considered significant.

## Results

### Timeline of the whole study and the baseline characteristics of all subjects

**Figure 1.**
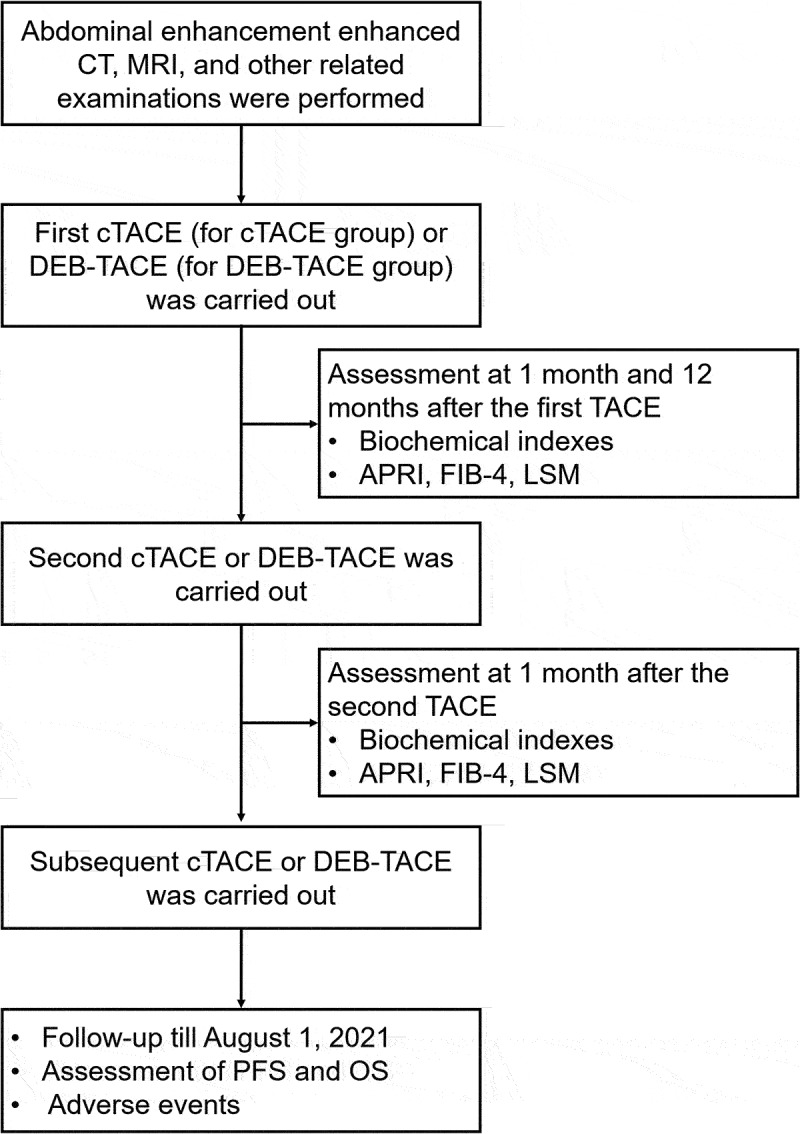
Study process.

To clarify the detailed process of this study, a timeline was presented (Figure 1). There were 62 HCC patients in the DEB-TACE group with a mean age of 57.1 ± 11.3 years and 59 patients in the cTACE group with a mean age of 56.8 ± 8.3 years (Table 1). The DEB-TACE group consisted of 11 (17.7%) females and 51 (82.3%) males; meanwhile, the cTACE group contained 8 (13.6%) females and 51 (86.4%) males. Notably, all baseline characteristics were not different between the DEB-TACE group and the cTACE group, including age, sex, ECOG PS, etiology, tumor distribution, largest nodule size, tumor capsule, portal vein invasion, splenomegaly, Child-Pugh stage, BCLC stage, and AFP (all *P* > .050). The detailed characteristics of these two groups were shown in Table 1.

**Table 1.** Baseline characteristics.

Items	DEB-TACE group (N = 62)	cTACE group (N = 59)	*P* value
Age (years), mean ± SD	57.1 ± 11.3	56.8 ± 8.3	0.869
Gender, n (%)			0.527
Female	11 (17.7)	8 (13.6)	
Male	51 (82.3)	51 (86.4)	
ECOG PS, n (%)			0.353
0	25 (40.3)	19 (32.2)	
1	37 (59.7)	40 (67.8)	
Etiology, n (%)			0.841
HBV infection	47 (75.8)	42 (71.2)	
HCV infection	5 (8.1)	6 (10.2)	
Others	10 (16.1)	11 (18.6)	
Tumor distribution, n (%)			0.322
≤3	27 (43.5)	31 (52.5)	
>3	35 (56.5)	28 (47.5)	
Largest nodule size (cm), mean ± SD	9.4 ± 4.0	9.6 ± 3.7	0.776
Tumor capsule, n (%)			0.125
No	37 (59.7)	43 (72.9)	
Yes	25 (40.3)	16 (27.1)	
Portal vein invasion, n (%)			0.314
No	33 (53.2)	26 (44.1)	
Yes	29 (46.8)	33 (55.9)	
Splenomegaly, n (%)			0.773
No	30 (48.4)	27 (45.8)	
Yes	32 (51.6)	32 (54.2)	
Child‒Pugh stage, n (%)			0.284
A	28 (45.2)	21 (35.6)	
B	34 (54.8)	38 (64.4)	
BCLC stage, n (%)			0.253
B	17 (27.4)	11 (18.6)	
C	45 (72.6)	48 (81.4)	
AFP, n (%)			0.813
<200 ng/mL	36 (58.1)	33 (55.9)	
≧200 ng/mL	26 (41.9)	26 (44.1)	

DEB-TACE, drug-eluting bead transarterial chemoembolization; cTACE, conventional transarterial chemoembolization; SD, standard deviation; ECOG PS, Eastern Cooperative Oncology Group Performance Status; HBV, hepatitis B virus; HCV, hepatitis C virus; BCLC, Barcelona Clinic Liver Cancer; AFP, alpha fetoprotein.

### Comparison of liver function indexes between two groups during the 1-year follow-up

**Figure 2.**
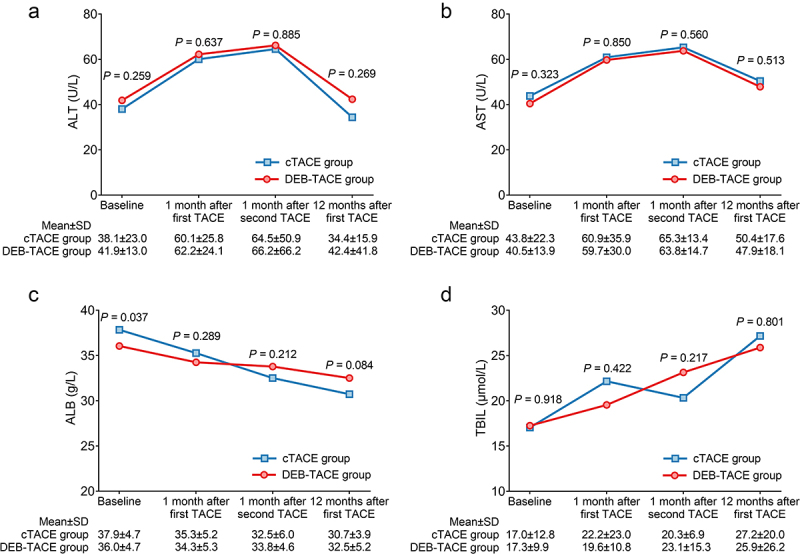
Liver function indexes between the DEB-TACE and cTACE groups were not different. Comparison of ALT (a), AST (b), ALB (c), and TBIL (d) at baseline, 1 month after the first TACE, 1 month after the second TACE, and 12 months after the first TACE between the DEB-TACE group and the cTACE group in HCC patients.

ALT (Figure 2a) and AST (Figure 2b) at baseline, 1 month after the first TACE, 1 month after the second TACE, and 12 months after the first TACE were not different between the DEB-TACE group and the cTACE group (all *P* > .050). ALB at baseline (*P* = .037) was decreased in the DEB-TACE group compared to the cTACE group, while ALB at 1 month after the first TACE, 1 month after the second TACE, and 12 months after the first TACE was not different between those two groups (all *P* > .050, Figure 2c). In addition, TBIL at baseline, 1 month after the first TACE, 1 month after the second TACE, and 12 months after the first TACE was not different between the two groups (all *P* > .050, Figure 2d).

### Comparison of HA, PC-III, IV-C, and LN between two groups during the 1-year follow up

**Figure 3.**
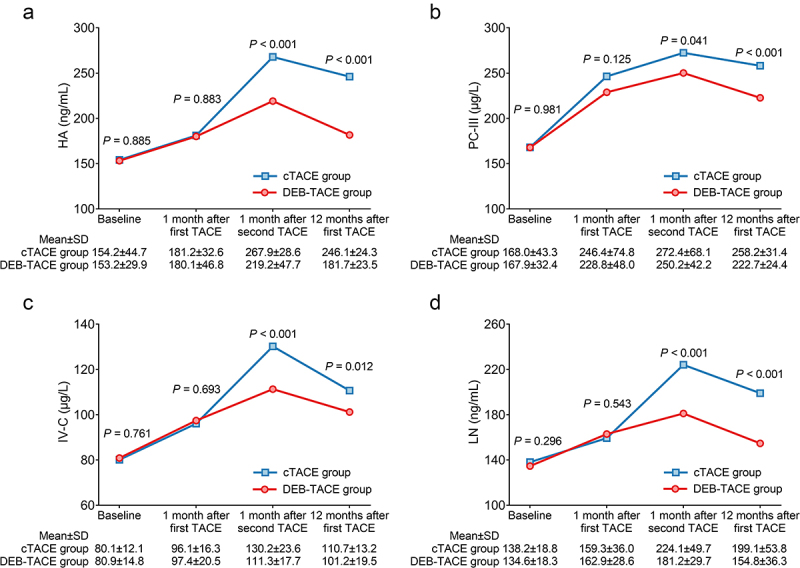
HA, PC-III, IV-C, and LN declined after treatment in the DEB-TACE group (vs. cTACE). Comparison of HA (a), PC-III (b), IV-C (c), and LN (d) at baseline, 1 month after the first TACE, 1 month after the second TACE, and 12 months after the first TACE between the DEB-TACE group and the cTACE group in HCC patients.

HA at baseline (*P* = .885) and at 1 month after the first TACE (*P* = .883) was not different between the DEB-TACE group and the cTACE group, while HA at 1 month after the second TACE (*P* < .001) and at 12 months after the first TACE (*P* < .001) was decreased in the DEB-TACE group compared with the cTACE group (Figure 3a). PC-III at baseline (*P* = .981) and at 1 month after the first TACE (*P* = .125) did not vary between the two groups, whereas PC-III at 1 month after the second TACE (*P* = .041) and at 12 months after the first TACE (*P* < .001) was decreased in the DEB-TACE group compared with the cTACE group (Figure 3b). No difference in IV-C at baseline (*P* = .761) or at 1 month after the first TACE (*P* = .693) was seen between those two groups, but IV-C at 1 month after the second TACE (*P* < .001) and at 12 months after the first TACE (*P* = .012) was reduced in the DEB-TACE group compared with the cTACE group (Figure 3c). LN at baseline (*P* = .296) and at 1 month after the first TACE (*P* = .543) was not different between those two groups, while LN at 1 month after the second TACE (*P* < .001) and at 12 months after the first TACE (*P* < .001) was decreased in the DEB-TACE group compared with the cTACE group (Figure 3d).

### Comparison of APRI, FIB-4, and LSM between two groups during the 1-year follow-up

**Figure 4.**
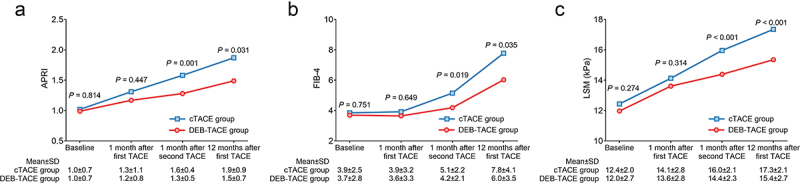
APRI, FIB-4, and LSM declined after treatment in the DEB-TACE group (vs. cTACE). Comparison of APRI (a), FIB-4 (b), and LSM (c) at baseline, 1 month after the first TACE, 1 month after the second TACE, and 12 months after the first TACE between the DEB-TACE group and the cTACE group in HCC patients.

APRI at baseline (*P* = .814) and at 1 month after the first TACE (*P* = .447) was not different between those two groups, while APRI at 1 month after the second TACE (*P* = .001) and at 12 months after the first TACE (*P* = .031) was decreased in the DEB-TACE group compared with the cTACE group (Figure 4a). FIB-4 at baseline (*P* = .751) and at 1 month after the first TACE (*P* = .649) did not vary between the two groups, while FIB-4 at 1 month after the second TACE (*P* = .019) and at 12 months after the first TACE (*P* = .035) was reduced in the DEB-TACE group compared with the cTACE group (Figure 4b). Similarly, the LSM at baseline (*P* = .274) and at 1 month after the first TACE (*P* = .314) was not different between the two groups, while the LSM at 1 month after the second TACE (*P* < .001) and at 12 months after the first TACE (*P* < .001) was reduced in the DEB-TACE group compared with the cTACE group (Figure 4c).

Further multivariate logistic regression model analysis revealed that DEB-TACE (vs. cTACE) was independently associated with a reduced occurrence of severe hepatic fibrosis at 12 months (odds ratio (OR): 0.215, 95% confidence interval (CI): 0.058–0.802, *P* = .022) (Table 2). Moreover, tumor distribution >3 (vs. ≤3) was independently related to the occurrence of severe hepatic fibrosis at 12 months after the first TACE (OR: 4.665, 95% CI: 1.320–16.495, *P* = .017).

**Table 2.** Factors related to severe hepatic fibrosis at 12 months after the first TACE by multivariate logistic regression model analysis*.

Items	*P* value	OR	95%CI	
			Lower	Upper
Treatment (DEB-TACE group vs. cTACE group)	0.022	0.215	0.058	0.802
Age (> 60 years vs. ≤ 60 years)	0.224	2.072	0.641	6.701
Gender (Male vs. Female)	0.553	0.617	0.125	3.040
ECOG PS (1 vs. 0)	0.339	2.247	0.427	11.820
Etiology (HBV infection vs. HCV infection or others)	0.212	2.630	0.575	12.026
Tumor distribution (>3 vs. ≤3)	0.017	4.665	1.320	16.495
Largest nodule size (>10 cm vs. ≤10 cm)	0.833	0.854	0.198	3.686
Tumor capsule (Yes vs. No)	0.332	1.771	0.559	5.617
Portal vein invasion (Yes vs. No)	0.779	1.252	0.261	6.005
Splenomegaly (Yes vs. No)	0.727	0.804	0.237	2.730
Child‒Pugh stage (B vs. A)	0.381	1.725	0.509	5.850
BCLC stage (C vs. B)	0.515	1.937	0.264	14.205
AFP (≧200 ng/mL vs. <200 ng/mL)	0.363	0.548	0.150	2.005
Number of TACE (>3 vs. ≤3)	0.062	0.269	0.068	1.067

TACE, transarterial chemoembolization; OR, odds ratio; CI, confidence interval; DEB-TACE, drug-eluting bead transarterial chemoembolization; cTACE, conventional transarterial chemoembolization; ECOG PS, Eastern Cooperative Oncology Group Performance Status; HBV, hepatitis B virus; HCV, hepatitis C virus; BCLC, Barcelona Clinic Liver Cancer; AFP, alpha fetoprotein. * Severe hepatic fibrosis was defined as LSM 12 months after the first TACE ≥17.5.

### Subgroup analyses of liver function and hepatic fibrosis indexes in patients with different etiologies

In patients with HBV infection, ALB at 12 months after the first TACE (*P* = .024) was elevated in the DEB-TACE group compared with the cTACE group, whereas HA (*P* < .001), PC-III (*P* < .001), IV-C (*P* < .001), LN (*P* < .001), and LSM (*P* = .001) at 12 months after the first TACE were reduced in the DEB-TACE group compared with the cTACE group. Concerning patients with HCV infection or others, only HA (*P* < .001), PC-III (*P* = .002), LN (*P* = .031), and APRI (*P* = .022) declined in the DEB-TACE group compared to the cTACE group (**Supplementary Table 1**).

### Comparison of survival profiles between two groups

**Figure 5.**
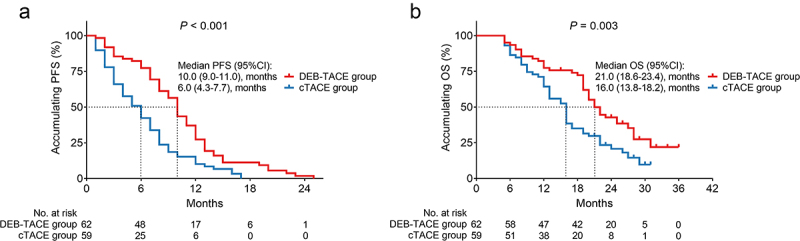
PFS and OS were prolonged in the DEB-TACE group (vs. cTACE). Comparison of PFS (a) and OS (b) between the DEB-TACE group and cTACE group in HCC patients.

Patients in the DEB-TACE group achieved prolonged PFS (*P* < .001, Figure 5a) and OS (*P* = .003, Figure 5b) compared to patients in the cTACE group. Specifically, the respective median PFS (95% CI) of patients in the DEB-TACE group and cTACE group was 10.0 (9.0–11.0) months and 6.0 (4.3–7.7) months, respectively. Furthermore, the median OS (95% CI) reached 21.0 (18.6–23.4) months in the DEB-TACE group and 16.0 (13.8–18.2) months in the cTACE group.

Furthermore, in severe hepatic fibrosis patients, neither PFS (*P* = .114, **Supplementary Figure 1A**) nor OS (*P* = .128, **Supplementary Figure 1B**) was different between the two groups. While in non-severe hepatic fibrosis patients, PFS (*P* = .004, **Supplementary Figure 1C**) was prolonged in DEB-TACE group compared to cTACE group, and the OS (*P* = .137, **Supplementary Figure 1D**) was not varied between the two groups.

### Comparison of adverse events between two groups

Generally, DEB-TACE exhibited a similar safety profile to cTACE. The incidence of most adverse events was not different between the DEB-TACE group and cTACE group, including fever (total: 41.9% vs. 54.2%, *P* = .176), pain (total: 41.9% vs. 49.2%, *P* = .425), constipation (total: 22.6% vs. 20.3%, *P* = .764), liver abscess (total: 1.6% vs. 3.4%, *P* = .613), and gastrointestinal hemorrhage (total: 0.0% vs. 1.7%, *P* = .488), except that the incidence of nausea and vomiting was decreased in the DEB-TACE group compared to the cTACE group (total: 21.0% vs. 55.9%, *P* < .001) (Table 3). Moreover, grade 3–4 adverse events were infrequent in the DEB-TACE group, including 4 (6.5%) pain cases and 1 (1.6%) constipation case.

**Table 3.** Comparison of adverse events between the two groups.

Items	DEB-TACE group (N = 62)			cTACE group (N = 59)			*P* value*
	Total	Grade 1–2	Grade 3–4	Total	Grade 1–2	Grade 3–4	
Fever	26 (41.9)	26 (41.9)	0 (0.0)	32 (54.2)	30 (50.8)	2 (3.4)	0.176
Nausea and vomiting	13 (21.0)	13 (21.0)	0 (0.0)	33 (55.9)	33 (55.9)	0 (0.0)	<0.001
Pain	26 (41.9)	22 (35.5)	4 (6.5)	29 (49.2)	23 (39.0)	6 (10.2)	0.425
Constipation	14 (22.6)	13 (21.0)	1 (1.6)	12 (20.3)	12 (20.3)	0 (0.0)	0.764
Liver abscess	1 (1.6)	1 (1.6)	0 (0.0)	2 (3.4)	2 (3.4)	0 (0.0)	0.613
Gastrointestinal hemorrhage	0 (0.0)	0 (0.0)	0 (0.0)	1 (1.7)	1 (1.7)	0 (0.0)	0.488

DEB-TACE, drug-eluting bead transarterial chemoembolization; cTACE, conventional transarterial chemoembolization.

*Test for total adverse events.

## Discussion

Hepatic fibrosis is considered a healing response to liver injury and is closely related to the treatment outcomes of HCC patients.^10^ In detail, patients with a lower hepatic fibrosis degree frequently recover sooner from the treatment, and they tend to have more subsequent treatment choices. Previously, only one study compared the impact of DEB-TACE and cTACE on hepatic fibrosis in treating HCC patients, which showed that IV-C and PC-III at 6 months after TACE were not different between the DEB-TACE group and the cTACE group, while LN and HA at 6 months after TACE were slightly lower in the DEB-TACE group than in the cTACE group.^25^ While in the current study, the four serologic fibrosis indexes (HA, PC-III, IV-C, LN) at 1 month after the second TACE and at 12 months after the first TACE were all decreased in the DEB-TACE group compared with the cTACE group, whereas these indexes at baseline and at 1 month after the first TACE were not different between the two groups. To further investigate the impact of DEB-TACE on hepatic fibrosis, APRI, FIB-4, and LSM were also determined, which showed similar trends to the above serologic fibrosis indexes. Additionally, DEB-TACE (vs. cTACE) was independently associated with a reduced occurrence of severe hepatic fibrosis at 12 months. Possible explanations might be as follows: (1) The previous study only determined the serologic fibrosis factors within 6 months, while the effects of DEB-TACE on avoiding severe hepatic fibrosis gradually appeared in the long term.^25^ Additionally, this study detected the fibrosis indexes at 12 months after the first TACE. Therefore, the decrease in hepatic fibrosis in the DEB-TACE group (vs. the cTACE group) was more obvious in this study than in a previous study. (2) Unlike the flowable lipiodol, DEB-TACE avoided drug leakage by releasing drugs in the targeted region, which further alleviated liver injury.^26^ Chronic liver injury is one of the leading causes of hepatic fibrosis.^27^ Consequently, DEB-TACE (vs. cTACE) prevented severe hepatic fibrosis in treating intermediate- or advanced-stage HCC patients. Moreover, it was observed that liver function indexes were not differed between DEB-TACE and cTACE, which could be explained by that: Liver function indexes were influenced by multiple factors, including hepatic fibrosis, inflammation, etc, hence, liver function indexes might not show the same variation as hepatic fibrosis indexes in some certain conditions (such as S_3_, S_4_ stage hepatitis B). Besides, number of TACE >3 (vs. ≤3) exhibited a correlating trend with decreased severe hepatic fibrosis risk, whose probable explanation was as follows: Patients who underwent more TACE therapy might become more tolerant, whose hepatic fibrosis degree was attenuated. While further studies with a larger sample size were necessary to validate this hypothesis.

Currently, the pleasing survival profile has been noticed in several studies.^19,20,28^ For instance, a meta-analysis study suggests that DEB-TACE exhibits improved PFS and OS over cTACE in treating HCC patients.^28^ In line with previous studies, the present study revealed that DEB-TACE realized prolonged PFS (median (95% CI): 10.0 (9.0–11.0) months vs. 6.0 (4.3–7.7) months, *P* < .001) and OS (median (95% CI): 21.0 (18.6–23.4) months vs. 16.0 (13.8–18.2) months, *P* = .003) compared to cTACE. Possible reasons might be as follows: (1) DEB-TACE kept high concentrations of the chemotherapeutic agent in the target lesion; subsequently, the treatment efficacy was satisfying.^18^ (2) DEB-TACE realized sustained release of the drug within a period of time, which provided more stable efficacy compared with cTACE.^19^ (3) DEB-TACE had better embolization effects because of its individualized size options. (4) DEB-TACE reduced the occurrence of severe hepatic fibrosis, while the latter factors induced HCC progression.^10^ Combining the above four aspects, DEB-TACE (vs. cTACE) realized prolonged PFS and OS in treating intermediate- or advanced-stage HCC patients. Additionally, it was worth mentioning that all patients in this study were treated with 100–300 μm CalliSpheres microspheres for the following reason: it was observed that CalliSpheres microspheres with a small particle size (100–300 μm) realized better short-term curative effects compared to the CalliSpheres microspheres with large particle size (300–500 μm) for the treatment of hepatocellular carcinoma patients.^29^

With respect to the safety profile of DEB-TACE, a previous study disclosed that the manifestations of postembolization syndrome or systemic toxicity were not different between the DEB-TACE group and the cTACE group.^30^ Consistent with a previous study, this study found that DEB-TACE exhibited a similar safety profile to cTACE, whose most adverse event incidences were not different, including fever (41.9% vs. 54.2%), pain (41.9% vs. 49.2%), constipation (22.6% vs. 20.3%), liver abscess (1.6% vs. 3.4%), and gastrointestinal hemorrhage (0.0% vs. 1.7%); only the incidence of nausea and vomiting was decreased in the DEB-TACE group compared to the cTACE group (21.0% vs. 55.9%). The possible reasons are listed as follows: (1) Unlike systemic therapy, TACE releases chemotherapeutic agents within the targeted lesion.^17^ Consequently, both DEB-TACE and cTACE displayed manageable adverse events. (2) As mentioned above, DEB-TACE prevented the leakage of chemotherapy drugs; meanwhile, nausea and vomiting were mainly caused by the chemotherapy drugs.^31^ Thus, the incidence of nausea and vomiting was decreased in the DEB-TACE group compared to the cTACE group.

Some inevitable limitations existed in this study. First, this study was not a randomized controlled trial; thus, confounding factors were difficult to avoid. Second, the current study applied DEB-TACE as a first-line treatment in HCC patients, while the impact of DEB-TACE on hepatic fibrosis in treating HCC patients with TACE history was unknown. Third, the surgical experience of each physician was different, which would also affect the efficacy of TACE.

Collectively, DEB-TACE displays attenuated hepatic fibrosis progression and noninferior tolerance compared to cTACE in treating intermediate- or advanced-stage HCC patients to some extent, which needs further verification in large-scale randomized controlled trials.

## Supplementary Material

Supplemental Material

## Data Availability

The authors confirm that the data supporting the findings of this study are available within the article and its supplementary materials.
