# Supplementary material for: The impact of drug-eluting bead (vs. conventional) transarterial chemoembolization on hepatic fibrosis in treating intermediate or advanced hepatocellular carcinoma
Source: Cancer Biol Ther. 2023 Feb 7;24(1):2166335. doi: 10.1080/15384047.2023.2166335 (PMC9928450; doi:10.1080/15384047.2023.2166335)
Supplement: Supplemental Material [file KCBT_A_2166335_SM7605.zip › Supplementary Table 1.docx]

**Supplementary table 1.** Subgroup analyses.

| Items | Etiology (HBV infection) | | | Etiology (HCV infection or others) | | |
| --- | --- | --- | --- | --- | --- | --- |
|  | DEB-TACE group | cTACE group | *P* value | DEB-TACE group | cTACE group | *P* value |
| **Indexes at 12 months after first TACE** | | |  |  |  |  |
| ALT (U/L), mean±SD | 45.1±47.3 | 34.9±17.1 | 0.289 | 34.8±19.6 | 33.2±13.3 | 0.822 |
| AST (U/L), mean±SD | 46.1±16.4 | 47.1±18.8 | 0.807 | 52.9±22.1 | 58.5±11.1 | 0.460 |
| ALB (g/L), mean±SD | 32.7±5.3 | 30.1±2.8 | 0.024 | 32.0±5.0 | 32.3±5.8 | 0.884 |
| TBIL (μmol/L), mean±SD | 26.1±27.7 | 28.2±22.9 | 0.752 | 25.1±22.4 | 24.6±10.3 | 0.939 |
| HA (ng/mL), mean±SD | 183.5±24.3 | 249.0±24.2 | <0.001 | 176.8±21.2 | 238.9±24.2 | <0.001 |
| PC-III (μg/L), mean±SD | 224.3±25.1 | 260.0±33.9 | <0.001 | 218.3±22.9 | 253.6±24.9 | 0.002 |
| IV-C (μg/L), mean±SD | 98.4±17.5 | 113.2±12.4 | <0.001 | 109.2±23.2 | 104.5±13.6 | 0.561 |
| LN (ng/mL), mean±SD | 154.4±35.9 | 199.6±56.1 | <0.001 | 155.8±39.0 | 197.9±50.5 | 0.031 |
| APRI, mean±SD | 1.5±0.7 | 1.6±0.8 | 0.340 | 1.6±0.8 | 2.4±0.9 | 0.022 |
| FIB-4, mean±SD | 5.3±2.6 | 6.5±2.9 | 0.089 | 8.0±4.9 | 10.9±5.0 | 0.170 |
| LSM (kPa), mean±SD | 15.3±2.7 | 17.5±2.1 | 0.001 | 15.4±2.6 | 17.0±2.1 | 0.117 |

HBV, hepatitis B virus; HCV, hepatitis C virus; DEB-TACE, drug-eluting beads transarterial chemoembolization; cTACE, conventional transarterial chemoembolization; TACE, transarterial chemoembolization; ALT, alanine aminotransferase; SD, standard deviation; AST, aspartate aminotransferase; ALB, albumin; TBIL, total bilirubin; HA, hyaluronic acid; PC-III, procollagen III; IV-C, collagen IV; LN, laminin; APRI, aspartate aminotransferase to platelet ratio index; FIB-4, fibrosis-4; LSM, liver stiffness measurement.
