# Supplementary figures and images for: The impact of drug-eluting bead (vs. conventional) transarterial chemoembolization on hepatic fibrosis in treating intermediate or advanced hepatocellular carcinoma
Source: Cancer Biol Ther. 2023 Feb 7;24(1):2166335. doi: 10.1080/15384047.2023.2166335 (PMC9928450; doi:10.1080/15384047.2023.2166335)

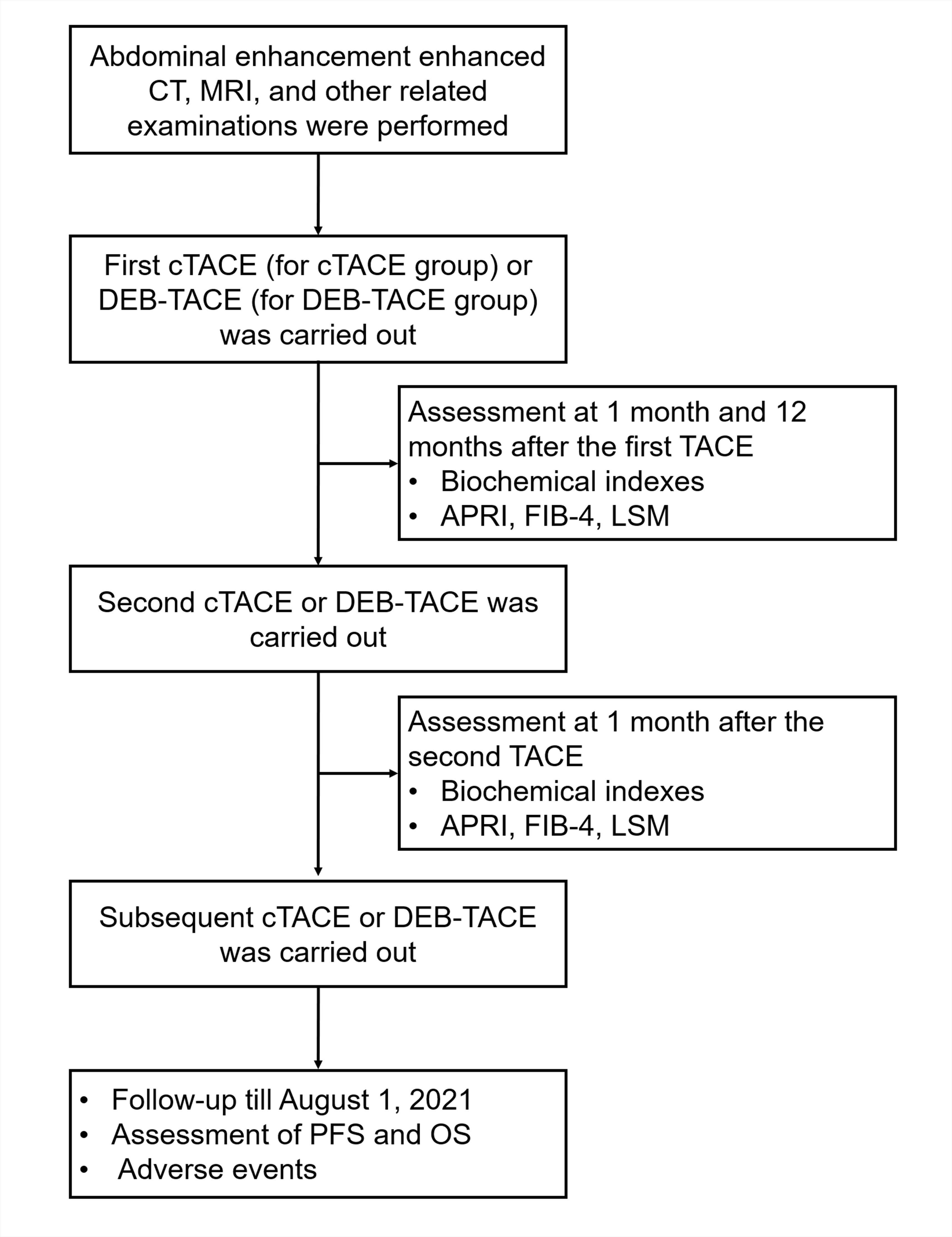

Supplement: Supplemental Material [file KCBT_A_2166335_SM7605.zip › Supplementary Figure 1.tif]
